# Editorial: Understanding violence: new data and theory

**DOI:** 10.3389/fsoc.2026.1837771

**Published:** 2026-04-30

**Authors:** Anthony Ellis, Simon Winlow, Daniel Briggs

**Affiliations:** 1 School of Social and Political Sciences, University of Lincoln, Lincoln, United Kingdom; 2 Humanities and Social Sciences, Northumbria University, Newcastle upon Tyne, United Kingdom

**Keywords:** structural violence, symbolic violence, systemic violence, violence, violent behavior

Violence terrifies and fascinates in equal measure. It is a “modern obsession,” “constantly in the news” and “ever present in popular entertainment” (Bessel, 2015: p. 1). This special issue brings together several contributions that engage with violence in physical, structural and symbolic forms within diverse geographical and cultural contexts spanning Southern and Northern America to Western and Eastern Europe. The nature and extent of violence globally is the subject of considerable debate and contestation amongst scholars; this Research Topic seeks to offer an important contribution to ongoing contemporary debates.

The twentieth century came to be regarded as “the long century of violence” (Keane, 1996). It was, of course, scarred by industrialized slaughter and the development of weapons capable of wiping out life on planet earth. The twenty-first century, by contrast, appears to have seen much less violence. Statistical measures suggest that violence is declining in many regions of the world. (Pinker 2012) has claimed that violence has been declining steadily for much of the modern age and encourages us to disconnect from the daily media spectacle of violence to focus instead on its statistically measured occurrence, which tells us we occupy possibly “the most peaceable era” in history (Pinker, 2012: p. xxi).

Findings from the United Nations' periodic study of global homicide (UNODC, 2023) lends some support to Pinker's influential thesis: the overall global homicide rate has declined during the past decade and is forecast to decline further by 2030. However, the current picture for homicide remains quite volatile; some regions experience consistently low and declining rates, while others report sudden and dramatic surges.

While Pinker suggests that the “better angels” of human nature now routinely triumph over the bad, an accurate rendering of human life in the twenty-first century demands a more careful and considered approach. In an era overshadowed by the reemergence of the threat of nuclear war, the suggestion we are living through a period of sustained “peace” requires critical appraisal. Conditions of famine, poverty, and injurious inequality in circumstances where such things are objectively avoidable, have been suggested to be constitutive of structural violence (Galtung, 1969). In seeking to push scholars to think beyond traditional conceptions of violence, (Zizek 2009) offered an influential tripartite framework incorporating subjective, symbolic and systemic forms. The latter, he argues, is akin to the “dark matter” of Physics, as it forms a largely imperceptible background that shapes everyday experience. For Zizek, systemic violence is the violence that sustains our present way of life. Grotesque inequality, creeping oppression and vulgar exploitation are omnipresent and tacitly accepted, to the extent that they constitute the normative experience against which we measure spectacular outbursts of subjective violence.

We might consider the gradual historical transformation of the drive to violence from the immediate physical realm into language and symbolism to be a broadly positive trend, but symbolic violence inflicts considerable damage upon individuals and communities on the receiving end. The inherent violence of competition and the possessive individualism that continues to gradually replace the modern age's fragile forms of community, severely test our ability to overcome our most compelling challenges. Despite the unshakable optimism of the system's propagandists, the scale and impact of what Zizek calls “subjective violence” still necessitates concerted academic attention. Despite claims of a decline in physical forms of violence across time, the UN has described violence against women and girls as “endemic” and estimates that globally 1 in 3 will be victimized during their lifetime. The UK, a nation with comparatively low homicide rates, saw this begin to increase in the years preceding the COVID-19 pandemic. Considering this, there is little doubt that much work still needs to be done.

This Research Topic should go some way to convincing readers that there remain many committed academics keen to shed light upon the nature, effects and meanings of contemporary violence. The contributions bring new data and theoretical advances to bear upon violence in its various forms. In response to the endemic scale of violence against women and girls globally, Dekeseredy makes an impassioned plea for the importance of Feminist approaches to gendered violence in an era where they have experienced considerable backlash. Reflecting Dekeseredy, Kujundzic's contribution provides, in the true spirit of Feminism, a voice for survivors of gendered violence that have been routinely overshadowed in post-conflict Croatia. Telford's contribution draws our attention to the systemic violence of neoliberal political economy in post-Brexit Britain. While some in the mainstream media continue to respond with shock and surprise at explosions of anger, hatred and xenophobia in Britain's “left-behind” communities, Telford identifies the insidious slow-burning effects of the violence of neoliberalism and its role in creating “absences” from which outbursts of visible violence emerge. Gibbs and Briggs shed light on the hidden injuries sustained by boys subject to the systemic violence of English professional football academies. Their contribution offers a glimpse of the rarely seen ruthless competition and injustice within the “beautiful game.” Through gathering the lived experiences of communities in Huanta in the Peruvian Andes, Huayhua Lévano et al. explore the roots of inter-generational resistance to persistent, insidious state violence. Finally, Miles and Azad complete this special issue through engaging critically with the “disposability” of service economy workers subject to cultural violence and physical attacks during their working lives.

As we continue to appraise a world scarred by historic, ongoing and emerging forms of violence and injustice, we hope you will join us in committing to the task of investigating these trends with the honesty and determination we need to fully understand and overcome them.
